# Caveolin-1 Single Nucleotide Polymorphism in Antineutrophil Cytoplasmic Antibody Associated Vasculitis

**DOI:** 10.1371/journal.pone.0069022

**Published:** 2013-07-19

**Authors:** Sourabh Chand, Julia U. Holle, Marc Hilhorst, Matthew J. Simmonds, Stuart Smith, Lavanya Kamesh, Peter Hewins, Amy Jayne McKnight, Alexander P. Maxwell, Jan Willem Cohen Tervaert, Stefan Wieczorek, Lorraine Harper, Richard Borrows

**Affiliations:** 1 Centre for Translational Inflammation Research, University of Birmingham, Birmingham, United Kingdom; 2 Human Genetics, Ruhr-University, Bochum, Germany; 3 Division of Clinical and Experimental Immunology, Maastricht University Medical Center, Maastricht, The Netherlands; 4 Oxford Centre for Diabetes, Endocrinology and Metabolism, University of Oxford, Oxford, United Kingdom; 5 Department of Nephrology, Queen Elizabeth Hospital Birmingham, Birmingham, United Kingdom; 6 Regional Nephrology Unit, Belfast City Hospital, Belfast, Northern Ireland; University of Sao Paulo Medical School, Brazil

## Abstract

**Objective:**

Immunosuppression is cornerstone treatment of antineutrophil cytoplasmic antibody associated vasculitis (AAV) but is later complicated by infection, cancer, cardiovascular and chronic kidney disease. Caveolin-1 is an essential structural protein for small cell membrane invaginations known as caveolae. Its functional role has been associated with these complications. For the first time, caveolin-1 (*CAV1*) gene variation is studied in AAV.

**Methods:**

*CAV1* single nucleotide polymorphism rs4730751 was analysed in genomic DNA from 187 white patients with AAV from Birmingham, United Kingdom. The primary outcome measure was the composite endpoint of time to all-cause mortality or renal replacement therapy. Secondary endpoints included time to all-cause mortality, death from sepsis or vascular disease, cancer and renal replacement therapy. Validation of results was sought from 589 white AAV patients, from two European cohorts.

**Results:**

The primary outcome occurred in 41.7% of Birmingham patients. In a multivariate model, non-CC genotype variation at the studied single nucleotide polymorphism was associated with increased risk from: the primary outcome measure [HR 1.86; 95% CI: 1.14-3.04; p=0.013], all-cause mortality [HR:1.83; 95% CI: 1.02-3.27; p=0.042], death from infection [HR:3.71; 95% CI: 1.28-10.77; p=0.016], death from vascular disease [HR:3.13; 95% CI: 1.07-9.10; p=0.037], and cancer [HR:5.55; 95% CI: 1.59-19.31; p=0.007]. In the validation cohort, the primary outcome rate was far lower (10.4%); no association between genotype and the studied endpoints was evident.

**Conclusions:**

The presence of a CC genotype in Birmingham is associated with protection from adverse outcomes of immunosuppression treated AAV. Lack of replication in the European cohort may have resulted from low clinical event rates. These findings are worthy of further study in larger cohorts.

## Introduction

Antineutrophil cytoplasmic antibody (ANCA)-associated small-vessel vasculitides (AAV) represent a heterogeneous group of clinical syndromes manifesting in multisystem disorders. Despite success in improving patient life expectancy, there remains high mortality at 5 years (28% in one study) and significant morbidity associated with complications of the disease and its treatment such as infection, cardiovascular disease, malignancy and chronic kidney disease [1].

Caveolae are lipid raft plasma cell membrane invaginations of 50-100nm length and although ubiquitously distributed, are prominently found on fibroblasts, endothelial and epithelial cells. Caveolin-1 is an essential protein within caveolae and acts as an intracellular signalling pathway chaperone in human fibrotic, vascular and malignant diseases [2–4]. Caveolin-1 also affects macrophage-related clearance of infection [5,6].

Thus it is biologically plausible that variation within *CAV1*, the gene encoding caveolin-1 may play a role in the complications of AAV and its treatment. Our group has previously identified an association between a single nucleotide polymorphism (SNP) within *CAV1* (rs4730751) and renal transplant fibrosis and vascular disease in two independent kidney transplant cohorts. Subsequently, Testa et al showed this same SNP was associated with carotid arterial media thickness in renal replacement therapy (RRT) patients [7,8].

The purpose of this study was to investigate whether this *CAV1* gene variant was associated with clinical outcome in AAV. The primary outcome measure was chosen as a combined end-point of time to all-cause mortality and time to RRT, as this encompassed the increased risk of death from the co-morbidities associated with AAV and the progression of kidney disease (and interstitial fibrosis). Following preliminary results in an AAV cohort from Birmingham, UK, a combined replication AAV cohort from Germany and the Netherlands was studied to validate the findings.

## Methods

### Ethics Statement

The study was performed in accordance with the principles expressed in the Declaration of Helsinki. Respective National Ethics Committee approval was sought from: South Birmingham Regional Ethics Committee 0723, Ethics committee of the University of Lübeck AZ-06-087 and the Maastricht Local Ethics Committee 05-158. Study participants provided written informed consent and the data were analyzed anonymously.

### Patients

In the UK, 200 patients with AAV presenting to the nephrology service at Queen Elizabeth Hospital, Birmingham between 1979 and 2009, in whom genomic DNA had also been isolated as part of the clinical research program at the centre, were included. To limit the confounding effect of population stratification, only white patients (self-reported ethnicity) were included in the study. Patients were diagnosed as ANCA-associated vasculitis according to the European Medicines Agency algorithm, where a patient has a clinical diagnosis of either granulomatosis with polyangiitis (GPA) or microscopic polyangiitis (MPA), supported by either a positive ANCA assay or a diagnostic biopsy, and the absence of an alternative explanation as previously described [9]. Eight patients presenting during this period with a clinical diagnosis of eosinophilic granulomatosis with polyangiitis (EGPA) were not included as nearly 50% are ANCA-negative and treated in a different manner [9].

The replication Northern European cohort included patients from the Vasculitis Centre Luebeck/Bad Bramstedt, Germany and the Maastricht University Medical Centre+, the Netherlands. In this cohort genomic DNA was available for 596 white patients with GPA or MPA presenting between 1982 and 2009 to the rheumatology services of the respective centres. Twenty-seven patients with EGPA were not included.

ANCA testing was performed by indirect immunofluorescence (Birmingham) and ELISA methods (Germany and the Netherlands) depending on how the data was collected historically.

### Genotyping

Genotyping and phenotyping of the cohorts were performed independently across centres. *CAV1* SNP rs4730751 genotyping was performed using the same Taqman® assay (Applied Biosystems, Warrington, UK) [7].

### Outcome Measures

The primary outcome measure was a composite of time to all-cause mortality or time to RRT (either dialysis or kidney transplantation). Established RRT was considered when the patient required on-going dialysis beyond 3 months. Patients who initially were dialysis dependent and/or died prior to 3 months were not considered as established RRT for the purpose of this analysis. This time period was selected as this is when the majority of patients recover their renal function after initial dialysis dependence [10,11].

Secondary outcomes included time to all-cause mortality, time to RRT, time to death from infection, time to death from a vascular cause (ischaemic heart disease, heart failure, abdominal aortic aneurysm or cerebral vascular accident) and time to cancer development from AAV diagnosis. This data was retrieved from the prospectively maintained institutional database, and retrospectively corroborated by examining death certificate records. Non-melanoma skin cancers were excluded. In the Northern European cohort, whilst no dates of cancer onset were available, it was known if cancer had developed during the follow-up period.

The independent effect of genotype on outcome was assessed by adjusting for the following relevant clinical and demographic characteristics: age at diagnosis, gender, type of ANCA and serum creatinine level at presentation.

### Statistical Analysis

Data is shown as median (1st and 3^rd^ quartiles) unless otherwise indicated. Group comparisons were assessed using Mann-Whitney U and χ^2^ testing as appropriate. Cumulative events were analysed with Kaplan-Meier estimates, with the log-rank test used for intergroup comparison. Time-to-event analyses were performed using a Cox proportional hazards model. Categorical outcome data was analysed by logistic regression. Genotype distributions were assessed for concordance with Hardy-Weinberg equilibrium using a χ^2^ goodness-of-fit test with a type 1 error rate set at 5%. *CAV1* gene variation at rs4730751 and other relevant clinical and demographic characteristics were initially examined in a series of univariate analyses. Known variables that influence AAV outcomes as well as genotype were included in multivariate analysis (i.e. no selection process was used to remove these variables). In addition, an interaction term “*CAV1* genotype x diagnosis” was analysed to examine a differential effect of genotype depending on clinical diagnosis. A type 1 error rate of 5% (p≤0.05) was associated with statistical significance. SPSS software, version 18 (SPSS Inc., Chicago, Illinois) was used for analysis.

## Results

### Birmingham Cohort

**Figure 1:**
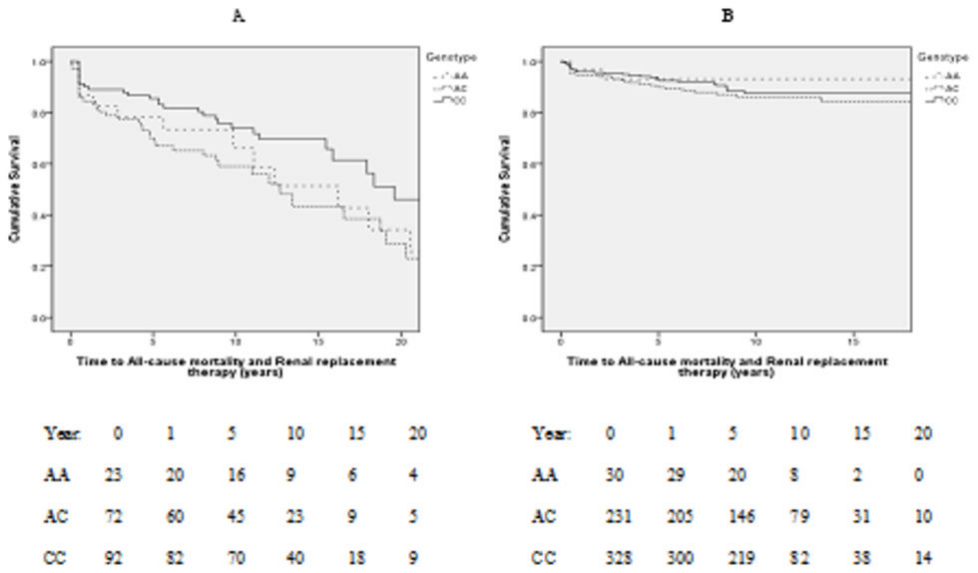
Kaplan-Meier analysis of time to all-cause mortality and renal replacement therapy in the Birmingham (A) and Northern European (B) cohort by genotype of *CAV1* SNP rs4730751. The number of patients at risk at separate time points is shown by year of follow-up. Graphic data shown to the last surviving 10% patients. p=0.022 (A) and p=0.427 (B).

In the Birmingham cohort, genomic DNA was successfully genotyped in 187 (>93%) patients. The *CAV1* SNP was within Hardy-Weinberg equilibrium bounds (p>0.05). Demographics of this cohort are shown in Table 1. The genotype of the patients at the studied locus was AA in 12.3% (23/187), AC in 38.5% (72/187), and CC in 49.2% (92/187). During a median follow-up of 110 months (interquartile range, 67-166 months), 58 patients died and 37 patients required RRT. There were 78 (41.7%) primary composite outcome events. Kaplan-Meier estimate of the primary combined outcome of time to all-cause mortality/RRT revealed significant differences between genotypes at rs4730751 (Figure 1A; p=0.022), with inferior outcomes in patients with non-CC genotype. Overall primary outcomes rates were 60.9% (14/23) for patients with genotype AA, 48.6% (35/72) for AC, and 31.5% (29/92) for CC genotypes. The Cox model examined patient genotype as non-CC (i.e. AA and AC combined) genotype versus CC genotype. A univariate association was found between genotype and time to all-cause mortality/RRT (non-CC vs. CC hazard ratio [HR]: 1.90; 95% confidence interval [CI], 1.19-3.02; p=0.007). This effect persisted in the multivariate analysis, where a similar HR was observed (non-CC vs. CC HR, 1.86; 95% CI, 1.14-3.08; p=0.013), adjusted for age at diagnosis, gender, type of ANCA and serum creatinine level at presentation.

**Table 1:** Demographics for Birmingham and Northern European cohorts are shown.

**Characteristic**	**Birmingham**	**Northern Europe**	**p value**
Number of patients	187	589	
Age, years	67 (56-77)	63 (52-71)	<0.001^1^
Age at diagnosis, years	58 (46-68)	54 (42-63)	0.003^1^
Male (%)	104 (56)	302 (51)	0.314^2^
*Genotype*			
AA (%)	23 (12)	30 (5)	0.001^2^
AC (%)	72 (39)	231 (39)	0.932^2^
CC (%)	92 (49)	328 (56)	0.130^2^
Granulomatosis with polyangiitis (%)	110 (59)	531 (90)	<0.001^2^
Microscopic polyangiitis (%)	77 (41)	58 (10)	<0.001^2^
Age at diagnosis, years	58 (46-68)	54 (42-63)	0.003^1^
Creatinine at presentation, µmol/L	182 (100-385)	90 (90-150)	<0.001^1^
Cytoplasmic (c) ANCA (%)	109 (58)	Not measured	
Perinuclear (p) ANCA (%)	71 (38)	Not measured	
Proteinase 3 (PR3) (%)	Not measured	438 (74)	
Myeloperoxidase (MPO) (%)	Not measured	97 (17)	
Negative (%)	7 (4)	54 (9)	

^1^ Mann-Whitney U test

^2^ Fisher’s exact test

### Secondary Analyses

After adjusting for age at diagnosis, gender, creatinine at presentation and type of ANCA, non-CC genotyped patients were at significantly increased risk for time to all-cause mortality, time to death from infection, time to death from a cardiovascular cause and time to cancer development from AAV diagnosis. There was some evidence for an increased risk of RRT for non-CC genotype, but this did not meet criteria for statistical significance (Table 2).

**Table 2:** Multivariate regression analysis of genotypes non-CC vs. CC for *CAV1* SNP rs4730751.

**Variable**	**HR**	**95% Confidence Interval**	**p value**
**Birmingham Cohort**			
Time to All-cause mortality	1.83	1.02-3.27	0.042
Time to Death from Vascular cause	3.13	1.07-9.10	0.037
Time to Death from Infection	3.71	1.28-10.77	0.016
Time to Cancer Development from AAV diagnosis	5.55	1.59-19.31	0.007
Time to Renal Replacement Therapy	1.79	0.86-3.73	0.119
**Northern Europe Cohort**			
Time to All-cause mortality	0.90	0.44-1.84	0.764
Time to Death from Vascular cause	1.33	0.27-6.67	0.729
Time to Death from Infection	0.24	0.28-2.00	0.196
Cancer	0.77*	0.28-2.17	0.625
Time to Renal Replacement Therapy	1.37	0.63-2.96	0.427

Times to event endpoint for both cohorts were adjusted for age at diagnosis, gender, creatinine at presentation and type of ANCA.

* Exact date of cancer onset during follow-up period not available, thus logistic regression analysis used, expressed as Odds Ratio

### Northern European Cohort

In the Northern European cohort, genomic DNA was successfully genotyped in 589 (>98%) patients. *CAV1* SNP rs4730751 was within Hardy-Weinberg equilibrium bounds (p>0.05). As shown in Table 1, there was a significantly lower AA genotype and higher CC genotype prevalence in Northern Europe as compared to the Birmingham cohort: AA genotype in 5.1% (30/589); AC genotype in 39.2% (231/589), and CC genotype in 55.7% (328/589). The Northern European cohort had over 90% of patients diagnosed with GPA as compared to fewer than 60% Birmingham. The Northern European cohort also had significantly lower creatinine values (suggesting a better kidney function) at presentation.

During a median follow-up of 89 months (interquartile range, 48-132 months), there were 33 deaths and 32 patients who required RRT. In all, there were 61 (10.4%) primary outcome events as compared to the 41.7% event rate in Birmingham described above. No association was seen between genotype and Kaplan-Meier estimates of time to primary outcome measure (Figure 1B; p=0.427). Overall primary outcome rates were 10.9% (2/30) for patients with AA genotype, 12.6% (29/231) for AC genotype, and 9.1% (30/328) for CC genotype. The Cox model showed no evidence of association between genotype and the primary outcome measure (non-CC genotype vs. CC genotype hazard ratio [HR]: 1.29; 95% confidence interval [CI], 0.78-2.13; p=0.319).

### Secondary Analyses

As shown in Table 2, no evidence for an association between genotype and any of the studied outcome measures was evident in the replication cohort.

### Interaction between *CAV1* genotype and Clinical Diagnosis

Due to current evidence of GPA and MPA being considered separate genetic disease entities, we next examined the influence of genotype for each diagnosis. The primary methodology for this was to examine a statistical interaction between *CAV1* genotype and diagnosis. In addition we performed a subgroup analysis for each of time to event outcome.

For all outcomes, the CC genotype was associated with lower risk. A significant interaction (last column of Table 3) between *CAV1* genotype and diagnosis was observed only for the ‘Renal Replacement Therapy’ outcome, whereby the influence of genotype was greater in patients with GPA. Although at first sight the results of the subgroup analyses seem to suggest a difference between diagnostic groups (second to last column of Table 3), this is likely a result of numbers in the study. The results of the interaction analyses (a more robust way of examining this issue) do not point to a differential effect of genotype between diagnoses.

**Table 3:** Cox regression analyses assessing the association of genotype upon the outcomes when the cohort is divided into diagnoses and then an assessment of the significance of interaction to address if the *CAV1* SNP genotype effect varied between diagnoses.

Time to Event	Diagnosis	Hazard Ratio (95% CI)	CC vs. non-CC P-value	Interaction P-value
All-cause mortality or RRT	GPA	0.56 (0.36, 0.86)	0.009	0.66
	MPA	0.67 (0.39, 1.15)	0.15	
All-cause mortality	GPA	0.74 (0.43, 1.29)	0.29	0.25
	MPA	0.47 (0.25, 0.90)	0.02	
Death from vascular cause	GPA	0.33 (0.11, 1.04)	0.06	0.95
	MPA	0.33 (0.10, 1.06)	0.06	
Death from infection	GPA	0.63 (0.24, 1.66)	0.35	0.49
	MPA	0.40 (0.15, 1.09)	0.07	
Cancer ^*)^	GPA	0.18 (0.04, 0.82)	0.03	0.62
	MPA	0.11 (0.01, 0.87)	0.04	
Renal Replacement Therapy	GPA	0.39 (0.20, 0.76)	0.006	0.04
	MPA	1.16 (0.54, 2.51)	0.70	

* Analysis performed on Birmingham data only, as time to cancer data was unavailable for the Northern European cohort.

## Discussion

This is the first study to evaluate the role of *CAV1* gene variation in determining clinical outcome in patients with AAV. In the Birmingham cohort, gene variation at the *CAV1* SNP rs4730751 was associated with the chosen combined primary end-point of time to all-cause mortality or RRT. In addition, secondary analyses showed an association between this SNP and all-cause mortality, death from infection, death from cardiovascular disease, and the development of cancer from AAV diagnosis.

These findings are plausible from previous studies in caveolin-1 biology, specifically with its role in vascular disease, sepsis, cancer and fibrosis. In particular, Testa et al found evidence of arterial remodelling was dependent on *CAV1* SNP rs4730751 genotype in patients requiring RRT [8]. Reduced caveolin-1 expression is also seen in vascular smooth muscle cells of patients with established atheromatous disease [12]. *Cav-1* deletion supressed macrophage phagocytosis with impaired bacterial clearance in murine models of sepsis, leading to increased mortality [6,13,14]. Caveolin-1 has also been widely associated as a prognostic marker in several cancers. Caveolin-1 down-regulation is found in human ovarian, lung and breast carcinomas [15]. In systemic sclerosis, caveolin-1 is thought to be anti-fibrotic by its effect on TGFβ signalling by receptor degradation [16]; as a model of accelerated fibrosis, renal allograft failure has been associated with *CAV1* SNP rs4730751 donor genotype [7].

The association between genotype and clinical outcome was not replicated in the Northern European cohort. There may be a number of reasons underlying this. The Northern European cohort appeared to have a lower expected event rate for an AAV cohort. Flossmann et al studied 535 patients with GPA or MPA as part of the European Vasculitis Study Group. Over a median follow-up of 5.2 years, there was a 25% mortality rate, with 32.3% of deaths from infection, 21.1% of deaths from cardiovascular cause, and a 9.3% cancer incidence [1]. Outcomes in the Birmingham cohort were similar, as over a median follow-up of 9.2 years the respective rates were 31.0%, 11.8%, 10.2% and 11.8%. In contrast, in the Northern European cohort over a median follow-up of 7.4 years, the respective rates were only 5.6%, 1.2%, 1.0% and 2.9%. The Northern European cohort was younger than that of Birmingham, and in particular had better kidney function at presentation. This likely reflects the “rheumatological” nature of the Northern European cohort versus the “nephrological” nature of the Birmingham cohort. Certainly kidney function at presentation is an important determinant of outcome in AAV, and this may explain the discrepancy between the cohorts, and the lack of association in the replication study. In addition, a difference in proportions of patients with GPA versus MPA was seen between the cohorts. Interestingly, we found some evidence that the influence of genotype differed between diagnoses for the time to requirement of renal replacement therapy, with a more marked effect in patients with a clinical diagnosis of GPA. This preliminary observation is relevant in light of emerging evidence that MPA and GPA are genetically distinct condition [9]. It should be acknowledged that the study design did not take in to account the varying immunosuppression protocols of the differing institutions, nor the severity of the disease activity. In addition, we acknowledge the possibility of ascertainment bias with regard to patient outcomes in this observational study. Finally, the issue of cryptic population stratification always exists in studies such as this, although the inclusion of only white patients goes some way to limit the confounding effect of this.

In summary, this is the first study to describe the association between *CAV1* variation and the outcomes of patients with ANCA associated vasculitis. This study suggests a relationship between the *CAV1* SNP rs4730751 and complications of AAV and its treatment, although the effect may be context-specific. Further investigation in future cohorts is warranted to establish whether this gene variant is of utility as a prognostic biomarker, either in isolation, or as part of a genetic biomarker panel.
